# A Randomized Controlled Intervention Trial with Danazol to Improve Telomeric and Fertility Parameters in Women with Diminished Ovarian Reserve: A Pilot Study

**DOI:** 10.1089/whr.2023.0013

**Published:** 2023-07-04

**Authors:** Isabel Córdova-Oriz, Graciela Kohls, Carlos Iglesias, Alba M. Polonio, Lucía Chico-Sordo, Mónica Toribio, Marcos Meseguer, Elisa Varela, Antonio Pellicer, Juan A. García-Velasco

**Affiliations:** ^1^ The Health Research Institute La Fe (IIS La Fe), IVI Foundation, Valencia, Spain.; ^2^ IVIRMA Madrid, Madrid, Spain.; ^3^ Laboratory of In Vitro Fertilization, IVIRMA Valencia, Valencia, Spain.; ^4^ School of Medicine, Department of Obstetrics and Gynecology, University of Valencia, Valencia, Spain.; ^5^ Department of Medical Specialties and Public Health, Rey Juan Carlos University, Madrid, Spain.; ^6^ IVIRMA Rome, Rome, Italy.

**Keywords:** Danazol, fertility, DOR, telomeres, TERRA, PBMCs

## Abstract

**Background:**

Most women who are treated at *in vitro* fertilization (IVF) clinics have trouble conceiving due to ovarian failure (OF), which seems to be associated to short telomeres and reduced or absent telomerase activity in their granulosa cells. Indeed, telomere pathways are involved in organ dysfunction. However, sexual steroids can stimulate the expression of the telomerase gene and have been successfully used to prevent telomere attrition. Thus, a strategy to improve IVF outcomes in women with OF could be telomerase reactivation using sexual steroids.

**Methods:**

We conducted a double-blind, placebo-controlled study. Patients with diminished ovarian reserve were randomized to Danazol or placebo for 3 months. We included patients with normal ovarian reserve in the study as untreated controls. Patients and controls underwent several ovarian stimulations (OSs). Telomere and IVF parameters were assessed.

**Results:**

We found that the mean telomere length in blood and the percentage of short and long telomeres were similar throughout the 3 months of treatment with Danazol. Remarkably, while the number of cells with one telomeric repeat-containing RNA (TERRA) focus decreased (*p* = 0.04) after the first month of Danazol treatment, the number of cells with 2 to 4 TERRA foci increased (*p* = 0.02). Regarding fertility, no differences were found in the antral follicle count. Interestingly, in OS performed after the trial, all Danazol-treated patients had a better MII oocyte rate compared to OS performed before the pilot study.

EudraCT number: 2018-004400-19.

**Conclusions:**

Danazol treatment seemed to affect telomere maintenance, since both the number of TERRA foci and the ratio of MII oocytes changed. However, further research is needed to confirm these results.

## Introduction

Increasingly delayed childbearing due to socioeconomic reasons is leading to natural ovarian aging and infertility problems.^1–3^ Ovaries have a limited time window during which they are fully functional, and they start to decay at around 35 years of age,^3^ with a decline in the quantity and quality of oocytes. This is the case of women with diminished ovarian reserve (DOR), who usually respond poorly to ovarian stimulation (OS) and have below-par *in vitro* fertilization (IVF) outcomes.^4^ A primary cause of aging and organ dysfunction is telomere attrition,^5^ which happens as cells divide, due to the fact that the DNA at the very end of the chromosome cannot be fully copied in each round of replication, resulting in gradual shortening.^6^

In mammals, telomeres consist of tandem repeats of the 5′-TTAGGG-3′ DNA sequence shaped by a protein complex called shelterin, which protects telomeres. In addition, telomeres confer identity to chromosome ends, to prevent them from being confused with DNA breaks.^7^ Telomere length (TL) in humans is ∼10 to 15 kb, with an attrition rate of about 70 base pairs per year^8^; thus, TL is a bona fide marker for biological aging.^5^ The telomeric C-strand is transcribed by RNA polymerase II, producing long noncoding RNAs which contain 5′-UUAGGG-3′ repeats, along with sequences that are present in the subtelomeric regions where transcription was initiated.^9,10^ These telomeric-repeat containing RNAs, called telomeric repeat-containing RNAs (TERRAs), can associate to telomeres to protect them,^11^ and are involved in TL regulation^12,13^ and telomerase recruitment.^10,14^

Several lines of evidence link telomeres to fertility decay. On one hand, women's ovarian reserve^15,16^ and anti-müllerian hormone (AMH) levels,^15^ a marker of ovarian reserve, decline steadily with age, increasing the risk of aging-associated diseases.^3^ On the other hand, shorter TL in blood is associated with lower oocyte quality^17^ and higher segregation errors^18^ and aneuploidy rates.^19–21^

In women with ovarian failure, shorter telomeres and reduced telomerase activity (TA) have been found in blood^22^ and granulosa cells (GCs).^22,23^ These women are usually exposed to lower doses of estrogens,^22^ which are known to activate telomerase.^24,25^ Aromatase-deficient mice show shortened TL and low telomerase levels along with ovarian dysfunction,^26^ and estrogen therapy reverses those symptoms.^26^ Thus, telomerase reactivation strategies may improve fertility through better telomere maintenance. Danazol, a synthetic steroid originally developed for endometriosis treatment, can act through the estrogen receptors, widely expressed in the body, including the ovaries, to bind with the telomerase promoter,^24^ activating telomerase expression *in vitro* and promoting telomere elongation.^27^

We hypothesized that treating DOR patients with Danazol could have beneficial effects for telomere maintenance and fertility outcomes. There are few studies using Danazol in clinical trials, and most of them did not include control or placebo groups, which provide valuable information of untreated and healthy age-matched women. Hence, both control groups were included in this study. In addition, our study provides retrospective data from IVF cycles that took place before and after the clinical trial. We found that Danazol treatment influenced the telomere pathway in peripheral blood mononuclear cells (PBMCs) and the MII oocyte rate in OS performed after treating the patients with Danazol.

## Materials and Methods

### Study design and participants

We conducted a pilot, double-blind, parallel-arm, and randomized controlled clinical trial with an inactive substance and a medication (Danazol, Sanofi-Aventis) at IVIRMA-Madrid between February 2020 and February 2022. In total, 19 women were recruited and divided into three groups with an average age of 39–41 (Table 1). The inclusion criteria required patients to be in good general health, as evidenced by their medical history, and to have a body mass index between 18 and 30 kg/m^2^. Exclusion criteria included steroid use or hormonal treatments up to 1 month before recruitment, to avoid bias with the study medicine. Women taking anticonvulsants, diabetes medication, anticoagulants or antihypertensives, as well as those who suffered irregular genital bleeding, or had thrombus or thromboembolic diseases, were also excluded.

**Table 1.** Characteristics of the Study Population

	NOR control group	Placebo-treated group	Danazol-treated group
Number of subjects, *n*	7	5	7
Age (years)			
Mean ± SD	39.5 ± 3.1	39.8 ± 2.7	41.2 ± 2.8
Median (Q1–Q3)	39.0 (36.0–43.0)	39.0 (37.5–42.5)	42.0 (38.0–43.0)
AMH levels (ng/mL)		0.5 ± 0.6	0.3 ± 0.4
Mean ± SD	3.1 ± 0.8	0.3 (0.1–1.1)	0.09 (0.05–0.8)
Median (Q1–Q3)	2.8 (2.5–3.5)	^*^p = 0.0002	^**^p = 0.0006
Primary infertility, *n* (%)	4 (57.1)	4 (80)	5 (71.4)
Secondary infertility, *n* (%)	3 (42.9)	1 (20)	2 (28.6)
Duration of infertility (years)			
Mean ± SD	1.0 ± 0.7	1.8 ± 1.0	0.9 ± 0.6
Median (Q1–Q3)	1.0 (0.5–2.0)	2.0 (1.0–2.5)	1.0 (0.5–1.5)
Reason for infertility, *n* (%)			
Advanced maternal age	5 (71.4)	4 (80.0)	5 (71.4)
Poor oocyte quality	0 (0)	0 (0)	2 (28.6)
Recurrent miscarriage	1 (14.2)	0 (0)	0 (0)
Premature ovarian failure	0 (0)	1 (20.0)	0 (0)
Other	1 (14.2)	0 (0)	0 (0)

Normality was calculated using the Shapiro-Wilk test.

^*^ *p*-Value calculated using a *t*-test to compare the mean AMH levels of the control and placebo-treated group.

^**^ *p*-Value calculated using the Mann–Whitney *U* test to compare the mean AMH levels of the control and danazol-treated group.

AMH, anti-müllerian hormone; n, number of individuals; NOR, normal ovarian reserve; SD, standard deviation.

**FIG. 1.**
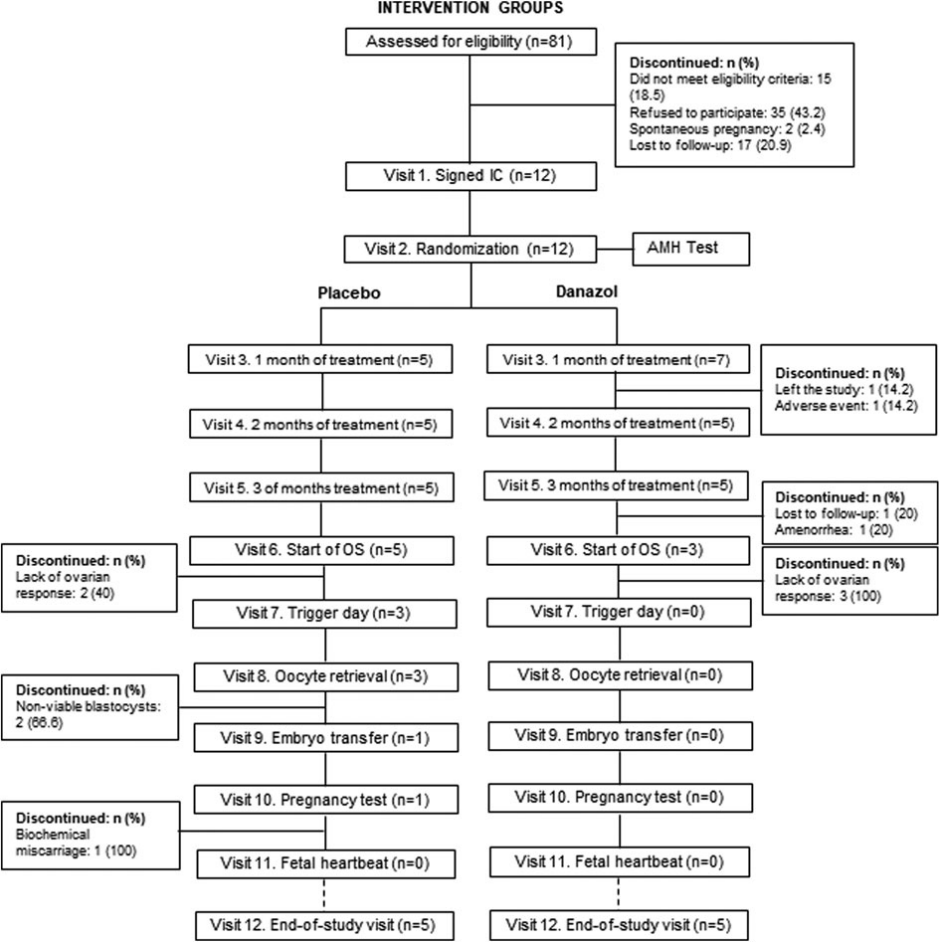
Intervention group flowchart. Flowchart detailing recruitment, randomization, treatment administration, and IVF procedures. The left panel shows the placebo-treated group, and the right panel shows the Danazol-treated group. AMH, anti-Müllerian Hormone; IC, informed consent; IVF, *in vitro* fertilization; *n*, number of individuals; OS, ovarian stimulation.

DOR patients (*n* = 12; AMH <2ng/mL) were randomized 1:1 to Danazol or placebo (Sanofi-Aventis) using computer-generated randomization. Gynecologists, embryologists, and the lead researcher were blinded to group allocation. The women participating in the study received the tablets (200 mg) in blisters (opaque and aluminum polyvinyl chloride) in the corresponding prepacked boxes each month of treatment. Once the data were declared clean and the database was locked, the data were released to the team for unblinding. DOR patients had up to 12 visits, which involved the revision of inclusion/exclusion criteria, treatment follow-up, and IVF procedures (Fig. 1). Patients took Danazol and placebo twice a day orally for 3 months.

Dose and treatment times in this study were lower compared to other studies^28^ to avoid side effects. Seven normal ovarian reserve (NOR) patients (AMH ≥2 ng/mL) were included as controls and did not take medication or placebo. Control patients had up to 9 visits for the revision of inclusion/exclusion criteria and IVF treatment (Supplementary Fig. S1).

### Outcomes

The primary endpoint was to reduce the accumulation of dysfunctional telomeres in GCs. Improving fertility parameters and analyzing the correlation between telomere factors in blood and GCs were secondary endpoints. Since the Danazol-treated patients cancelled their OS in the clinical trial due to poor response, it was not possible to obtain their GCs, so telomere factors were not measured in these cells. TL was measured in PBMCs, and fertility analysis focused on follicular development and oocyte yield. A retrospective study was included to compare IVF data before and after the clinical trial to understand the possible effects of Danazol treatment.

### Ethical approval

The pilot trial was approved on May 6, 2019, by the Ethics Committee of Puerta de Hierro University Hospital and on May 30, 2019, by the Spanish Agency of Medicines and Medical Devices. The study was registered in the European Union Drug Regulating Authorities Clinical Trials Database (EudraCT number: 2018-004400-19). In their first visit, all the participants signed an informed consent containing a detailed description of the exclusion and inclusion criteria (Fig. 1). A retrospective study (2203-MAD-038-MV code) was approved by the Ethics Committee of Puerta de Hierro University Hospital on April 28, 2022. The study was conducted in line with the principles outlined in the Declaration of Helsinki.

### Safety monitoring

The baseline health status of the patients included in each group was evaluated in the first and second visits (Fig. 1). IVIRMA gynecologists and nurses performed a physical examination, an electrocardiogram (to measure patients' heart rate and blood pressure), an AMH test, routine blood tests (total cell count, hemogram, and coagulation analysis), and a liver function test that measured several hepatic markers (aspartate aminotransferase, glutamic-pyruvic transaminase, and gamma-glutamyl transferase levels). The physical examination, blood tests, and liver function test were repeated every month during treatment (visits 3, 4 and 5; Fig. 1). Finally, blood tests and physical examinations were performed once more in the end-of-study visit (visit 12, Fig. 1).

Adverse clinical events were monitored in line with the regulations established by the Food and Drug Administration. The onset of malignant cancers has not been reported in clinical trials using Danazol and is not described as a side effect in the Danazol leaflet. In the control group, IVIRMA gynecologists and nurses performed the physical examination, the electrocardiogram, the blood tests, and the liver function test in visits 1, 2, and 3 and in the end-of-study visit (Supplementary Fig. S1).

### IVF procedures

Antral follicles (2–20 mm) were measured using a vaginal ultrasound (7.5 MHz) before and after the treatment (visits 2 and 5, respectively; Fig. 1) to assess changes in patients' ovarian reserve. In visit 6 (Fig. 1), intervention patients underwent OS with daily doses of follicle-stimulating hormone 225–300 IU (Bemfola, Gedeon Richter) and highly purified human menopause gonadotropin 75 IU (Ferring) following the Gonadotropin-releasing hormone GnRH antagonist protocol. As for the control group, they started OS on visit 3 (Fig. S1), with the same antagonist protocol. When at least three 17–18 mm follicles were observed, triptorelin 0.2 mL s.c. (Decapeptyl, Ipsen) was administered to induce final oocyte maturation (visit 6 for the intervention groups and visit 4 for the control group; Fig. 1 and Supplementary Fig. S1).

Oocytes were collected 36 hours later (visit 8 for the intervention groups and visit 5 for the control group; Fig. 1 and Supplementary Fig. S1). Mature oocytes (MII oocytes) were fertilized using an intracytoplasmic sperm injection and, in those cases when euploid blastocysts were obtained, a single embryo transfer was performed (visit 9 for the intervention groups and visit 6 for the control group; Fig. 1 and Supplementary Fig. S1). Women with negative results in the β-human chorionic gonadotropin test (Elecsys HCG+β Assay, Roche; visit 10 for the intervention groups and visit 7 for the control group), or women who had to cancel any of the above visits, had to proceed to the end-of-study visit (Fig. 1 and Supplementary Fig. S1).

After the end-of-study visit, once the retrospective study had been approved, we collected data for OS performed before and after the study to analyze the effect of Danazol on long-term IVF parameters. The ratio of MII oocytes was calculated dividing the number of MII oocytes by the number of follicles > than 12 mm in the same OS.

### Sample collection

The venipuncture site for blood sample collection was the antecubital fossa. For serum analysis (AMH and hepatic markers), 5 mL of blood was collected in tubes with gel serum separator (Sarstedt). For coagulation analysis, 5 mL of blood was collected in lithium heparin gel tubes (Sarstedt). For experimental analysis and hemograms, 5 mL of blood was collected in ethylenediaminetetraacetic acid (EDTA) tubes (Sarstedt). PBMCs were purified using a Ficoll gradient (Histopaque, Sigma), and fixed with methanol and acetic acid (3:1 ratio) and stored at 4°C until use for experimental analysis.

Blood samples for the experimental analysis of the intervention groups were collected in visits 2, 3, 4, and 5 (Fig. 1): pretreatment and after 1, 2, and 3 months of placebo or Danazol treatment. Blood samples for the experimental analysis of control patients were collected on visits 2 and 5 (Supplementary Fig. S1). Blood samples were taken from all patients to establish their baseline health status in the visits detailed previously, in the “Safety monitoring” section.

### Evaluation of baseline health status parameters and AMH levels

The samples were analyzed using chemiluminescence on the Cobas e411 bioanalyzer at IVIRMA Madrid. AMH levels were detected in serum using a sensitive Elecsys^®^ AMH Plus Assay (Roche), following the manufacturer's instructions, to estimate the ovarian reserve. Routine blood tests were analyzed using a BC-20s auto hematology analyzer (Mindray) at IVIRMA Madrid.

### High-throughput TL quantification by fluorescence *in situ* hybridization

High-throughput quantitative fluorescence *in situ* hybridization (HT Q-FISH) was performed on PBMCs as previously described in the study published in 2007 by Canela, Vera et al. Cells, fixed with a methanol and acetic acid solution (3:1) and adhered to 96-well plates (Greiner, Bio-One) with poly-L-Lysine (Sigma Aldrich), had a density of 200,000 cells per well. Cells were permeabilized with 0.1% porcine pepsin (Sigma Aldrich) for 10 minutes at 37°C and then dehydrated with increasing concentrations of EtOH (70%, 90%, and 100%) for 5 minutes at room temperature (RT). DNA was hybridized with a Cy3-labeled telomere probe [Cy3-(CCCTAA)_3_] (Panagene) in a hybridization solution (containing 70% deionized formamide, Ambion). The hybridization solution with no probe was added as a negative control.

After DNA denaturation, PBMCs were incubated with the probe at RT in the dark for 2 hours. Intensive washes with diluted formamide (Sigma Aldrich) were performed to remove the nonspecific probe.^29^ Nuclei were stained with 4′,6-diamidino-2-phenylindole at 1 μg/mL (DAPI, Invitrogen) and 1 μg/mL of antifading agent was added (Vectashield, Vector Laboratories). Telomeres from all blood samples were analyzed side by side on the same plate in duplicates (up to 60 samples can be included per plate).

### RNA FISH to measure TERRA

High-throughput RNA FISH was performed to analyze TERRA intensity levels in PBMCs as in previous studies.^11^ Approximately 200,000 per well were adhered to 96-well plates (Greiner, Bio-One) using poly-L-Lysine (Sigma Aldrich). PBMCs were permeabilized with 0.5% Triton X-100 (Sigma Aldrich) at RT for 1 hour and dehydrated with increasing concentrations of EtOH (70%, 80%, 95%, and 100%), previously cooled to 4°C. A telomere probe [Cy3-(CCCTAA)_3_] (Panagene) was diluted in a hybridization solution (containing 70% deionized formamide, Ambion) and heated at 95°C for 5 minutes.^11^ Cells were incubated with the probe at 37°C, overnight in the dark.

Finally, intensive washes with diluted formamide (Sigma Aldrich) and saline sodium citrate buffer (Panreac) were performed to remove the nonspecific probe. Nuclei were stained with DAPI at 1 μg/mL (Invitrogen) and 1 μg/mL of antifading agent was added (Vectashield; Vector Laboratories). Control cells treated with RNase A (Roche) were included (Supplementary Fig. S2). TERRA foci from all blood samples were analyzed side by side on the same plate in duplicates.

### Image acquisition

To analyze telomere Q-FISH and TERRA RNA FISH spot intensity, images were acquired using an Opera High-Content Screening System (PerkinElmer). We acquired 60 independent images per well. These images were analyzed with Acapella Software (PerkinElmer) as in previous studies.^30^ The Cy3 spot intensity values were exported to Excel spreadsheets. DAPI stain excitation was at 405 nm, and emission was between 430 and 470 nm. For Cy3 dye, excitation was at 561 nm, and the signal was detected between 579 and 679 nm. Without the telomeric probe, no telomeric signals were detected. The accumulation of short (15th Percentile) or long (85th Percentile) telomeres was calculated as in previous studies.^8^

### Statistical analysis

All statistical analyses were performed using GraphPad Prism software (version 8). Normality of the data was analyzed using the Shapiro-Wilk test. When two different study groups were compared, the *p*-values of normal data distributions were calculated using the Student's *t*-test. For non-normal data distribution, the *p*-values were calculated using the Mann–Whitney *U* test; *p*-values in the same study group (for instance, different visits) were calculated using a paired *t*-test. *p* < 0.05 was considered statistically significant.

## Results

### Recruitment

Eligible participants were recruited at IVIRMA Madrid from February 2020 to November 2021. Recruitment had to be interrupted from March 15 to June 21, 2020, due to the state of emergency declared in Spain because of the coronavirus disease 2019 (COVID-19) pandemic. Recruited patients continued to take the treatment and were medically monitored via telephone. Once recruitment was resumed, it progressed very slowly due to health restrictions that reduced the number of women attending reproduction clinics.^31^ COVID-19 hindered recruitment, leading to low participation and, ultimately, to the study ending sooner than expected.

### Baseline demographics

In total, 12 women with DOR were enrolled. Five patients were randomized to the placebo-treated group and seven to the Danazol-treated group. Seven women with NOR were recruited to act as a control group. Table 1 shows the main characteristics of each group. The mean age was similar in the three groups, with no significant differences (Table 1). Most patients showed primary infertility due to advanced maternal age (Table 1). AMH levels were statistically significantly higher (Table 1) in the control group (3.1 ± 0.8 ng/mL) compared to the placebo-treated (0.5 ± 0.6 ng/mL; *p* = 0.0002) and Danazol-treated group (0.3 ± 0.4 ng/mL; *p* = 0.0006). The intervention groups did not show statistically significant differences in AMH values (Table 1).

Two patients included in the Danazol-treated group abandoned the study, one due to mild adverse effects and the other due to reasons unrelated to the study (Fig. 1). The side effects recorded during the trial were all included in the package leaflet of the drug. All the patients in the placebo-treated and control group completed the study (Fig. 1).

### Analysis of the effects of Danazol on telomere maintenance in PBMCs

**FIG. 2.**
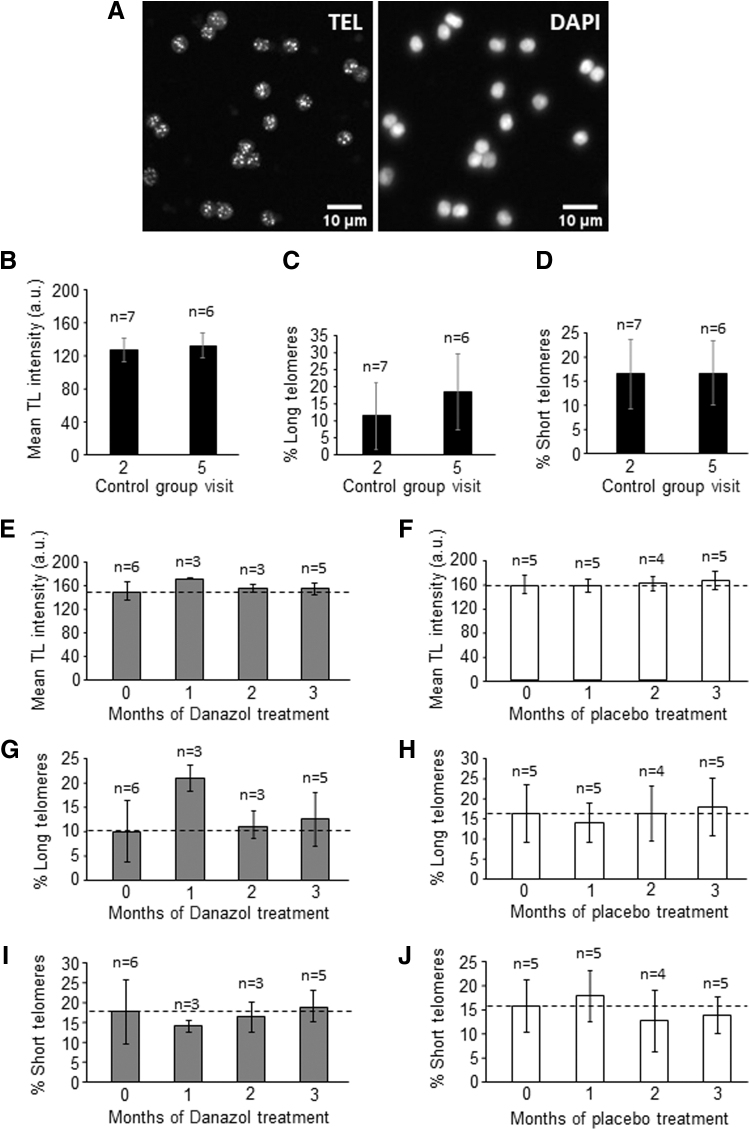
Analysis of TL in PBMCs. **(A)** The micrographs show representative images of telomere Q-FISH (white dots, left panel) and DAPI (right panel) in PBMCs. **(B)** Mean TL in PBMCs, measured using Q-FISH, in the control group on visits 2 and 5. **(C)** Percentage of long telomeres in PBMCs of the control group on visits 2 and 5. **(D)** Percentage of short telomeres in PBMCs of the control group on visits 2 and 5. **(E)** Mean TL in PBMCs, measured using Q-FISH, in the Danazol-treated group on the pretreatment visit (month 0) and after 1, 2, and 3 months of treatment. **(F)** Mean TL in PBMCs, measured using Q-FISH, in the placebo-treated group on the same months indicated in **E**. **(G)** Percentage of long telomeres in PBMCs of the Danazol-treated group on the same months indicated in E. **(H)** Percentage of long telomeres in PBMCs of the placebo-treated group on the same months indicated in E. **(I)** Percentage of short telomeres in PBMCs of the Danazol-treated group on the same months indicated in E. **(J)** Percentage of short telomeres in PBMCs of the placebo-treated group on the same months indicated in E. The error bars show the SD in all graphs. n: number of individuals analyzed. Scale bar, 10 μm. DAPI, 4′,6-diamidino-2-phenylindole; PBMCs, peripheral blood mononuclear cells; Q-FISH, quantitative fluorescence *in situ* hybridization; SD, standard deviation; TEL, telomeres; TL, telomere length.

To analyze whether Danazol had any effect on telomere maintenance, we first measured TL in PBMCs. There were no statistical differences in the mean TL of controls (both visits) or in the percentage of long and short telomeres (Fig. 2A–D). In women with DOR, there were no statistically significant changes in mean TL (Fig. 2E, F). Similarly, no changes were observed in the percentage of long telomeres in the Danazol- (Fig. 2G) or placebo-treated (Fig. 2H) group, or in the percentage of short telomeres in either group (Fig. 2I, J).

**FIG. 3.**
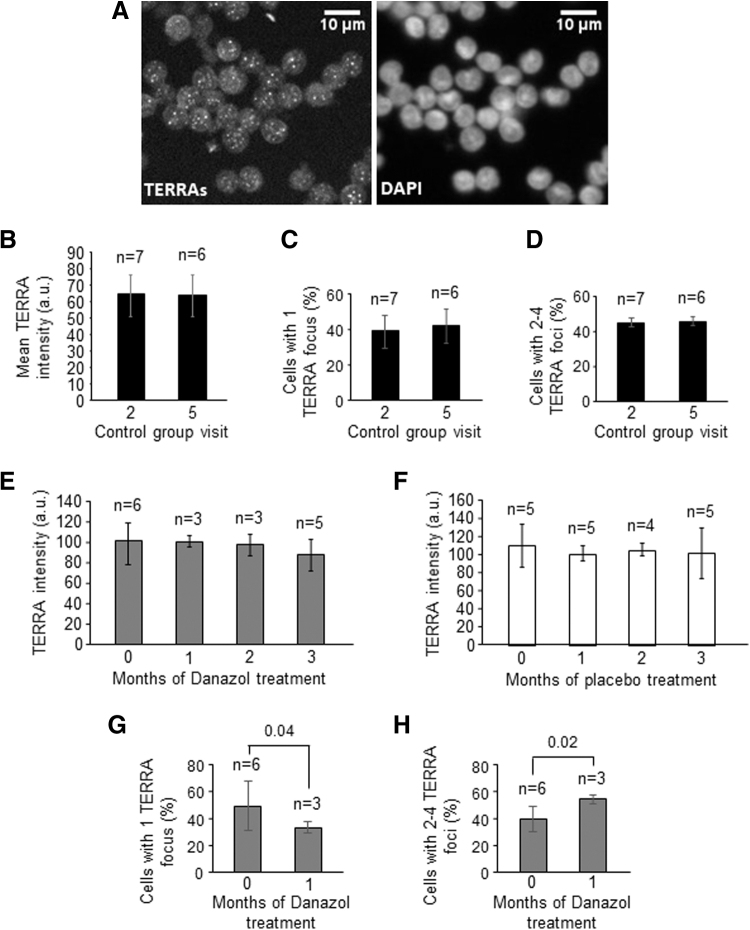
TERRA expression in PBMCs. **(A)** The micrographs show representative images of TERRAs (white spots, left panel) and DAPI (right panel). **(B)** Mean TERRA intensity levels, measured using RNA FISH, in the control group, on visits 2 and 5. **(C)** Percentage of cells with 1 focus, in the control group, on visits 2 and 5. **(D)** Percentage of cells with 2 to 4 TERRA foci, in the control group, on visits 2 and 5. **(E)** Mean TERRA signal intensity, measured using RNA FISH, in the Danazol-treated group on the pretreatment visit (month 0) and after 1, 2, and 3 months of treatment. **(F)** Mean TERRA signal intensity, measured using RNA FISH, in the placebo-treated group on the same months indicated in E. **(G)** The graphs show the percentage of cells with 1 TERRA focus in the Danazol-treated group in the pretreatment and 1 month post-treatment visit. **(H)** The graphs show the percentage of cells with 2 to 4 TERRA foci in the Danazol-treated group in the pretreatment and 1-month post-treatment visit. The error bars show the SD in all graphs. *n*: number of individuals analyzed. Scale bar, 10 μm. TERRA, telomeric repeat-containing RNA.

Because of the many important functions of TERRAs in telomere maintenance,^11^ we analyzed them in PBMCs. We found no differences in mean TERRA intensity (Fig. 3A, B) or in the number of TERRA foci per cell (Fig. 3C, D) in the control group, and RNase A-treated cells showed no TERRA signal (Supplementary Fig. S2). Likewise, in the Danazol-treated group, the mean TERRA intensity levels were similar before and during treatment (Fig. 3E). In the placebo-treated group, the mean TERRA intensity was also similar in all visits (Fig. 3F). In the Danazol-treated group, the percentage of cells with one TERRA focus decreased after the first month of treatment (Fig. 3G; *p* = 0.04) and remained stable up to 2 months of treatment (Supplementary Table S1; *p* = 0.01).

Conversely, the number of cells with 2 to 4 TERRA foci increased after 1 month of treatment (Fig. 3H; *p* = 0.02) and remained stable up to the second month of treatment (Supplementary Table S1; *p* = 0.03). There were no statistically significant changes in the percentages of cells with TERRA foci at any time in the control and placebo-treated group (Supplementary Table S1). The percentage of cells with more than five TERRA foci did not change in any group (Supplementary Table S1). Together, these results point to an effect of Danazol on telomere maintenance.

### Analysis of the effects of Danazol on fertility

The control group had higher AMH levels (Table 1) and, thus, a higher antral follicle count (AFC) (13.0 ± 3.1; Table 2). As for the intervention groups, no differences were found in the AFC before and after treatment (3.4 ± 1.1 vs. 4.2 ± 0.5 in the placebo-treated group, and 3.6 ± 3.5 vs. 3.2 ± 2.9 in the Danazol-treated group; Table 2). Right after intervention, patients underwent OS. All patients in the Danazol-treated group cancelled their first OS and oocyte retrieval due to a very low number of mature follicles (bigger than 12 mm in diameter) evaluated by ultrasound (Table 2). The placebo-treated group had higher AMH levels (Table 1) compared to the Danazol-treated group, and thus more mature follicles (Table 2). The number of follicles (total and >12 mm) and oocytes (total and MII) obtained in placebo-treated DOR patients was statistically significantly lower than in the control group (Table 2).

**Table 2.** Analysis of Fertility Parameters

	NOR control group	Placebo-treated group	Danazol-treated group
Number of subjects, *n*	7	5	5
AFC pre-treatment		3.4 ± 1.1	3.6 ± 3.5
Mean ± SD	13.0 ± 3.1	3.0 (2.5–4.5)	3.0 (0.5–7.0)
Median (Q1–Q3)	14.0 (11.0–16.0)	^*^p = < 0.0001	^**^p = 0.0007
AFC post-treatment			
Mean ± SD	Not treated	4.2 ± 0.5	3.2 ± 2.9
Median (Q1–Q3)		4.0 (4.0–4.7)	3.0 (0.5–6.2)
Initiated OS cycles, *n*	7	5	4
Cancelled cycles, *n* (%)	1 (14.3)	2 (40)	4 (100)
Follicular detection by ultrasound during OS			
Total follicles	14.1 ± 4.1	5.0 ± 3.8	1.5 ± 0.7
Mean ± SD	15.0 (13.0–17.0)	4.0 (2.0–8.5)	1.5 (1.0–2.0)
Median (Q1–Q3)		^*^p = 0.002	^**^p = [0.004]
Follicles >12 mm	12.7 ± 5.3	3.4 ± 3.6	0.5 ± 0.7
Mean ± SD	15.0 (6.0–17.0)	2.0 (0.5–7.0)	0.5 (0.0–1.0)
Median (Q1–Q3)		^*^p = 0.007	^**^p = 0.01
Number of oocytes			Oocyte retrieval cancelled
Total oocytes	12.3 ± 5.6	3.3 ± 3.2	x
Mean ± SD	13.0 (7.7–15.7)	2.0 (1.0–7.0)	
Median (Q1–Q3)		^*^p = 0.04	
MII oocytes	11.3 ± 5.3	2.6 ± 2.0	X
Mean ± SD	11.5 (7.0–14.7)	2.0 (1.0–5.0)	
Median (Q1–Q3)		^*^p = 0.03	

Normality was calculated using the Shapiro-Wilk test.

^*^ *p*-Value calculated using a *t*-test to compare the parameters analyzed in the control and placebo-treated group.

^**^ *p*-Value calculated using a *t*-test to compare the parameters analyzed in the control and danazol-treated group.

AFC, antral follicle count; OS, ovarian stimulation.

**FIG. 4.**
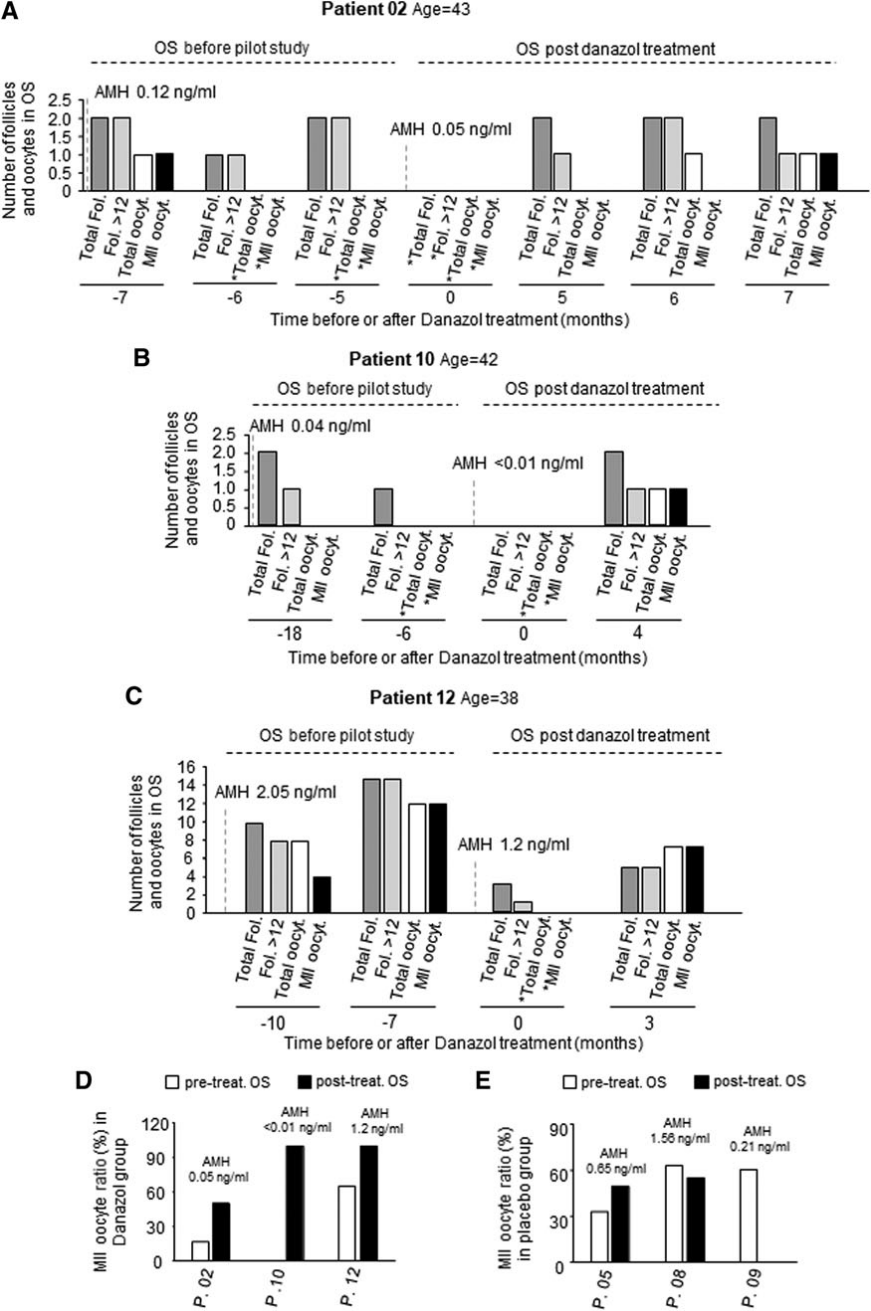
Follicle and oocyte analysis in the Danazol-treated group. **(A)** Number of follicles and oocytes produced by patient 02 in several OS cycles in the months specified before the start of the clinical trial and other OS cycles performed after the end of Danazol treatment. **(B)** Same data as in A, but for patient 10. **(C)** Same data as in A, but for patient 12. In all graphs, month “0” indicates the first OS cycle performed right after Danazol treatment. Dark gray bars represent total follicles after OS; light gray bars, follicles larger than 12 mm; white bars, total oocytes retrieved in puncture after OS; and black bars, MII oocytes obtained after OS. * Indicates absence of data due to either loss of contact with the patient or cancellation of the oocyte retrieval. **(D)** The percentage of mature oocytes with regard to the number of follicles >12 mm in diameter for patients 02, 10, and 12 in the Danazol-treated group. White bars show data form pretreatment OS cycles and black bars show data from post-treatment OS cycles. Please note that the OS cycle performed right after Danazol treatment was not considered in the analysis due to the androgenic effect of Danazol. **(E)** The percentage of mature oocytes with regard to the number of follicles >12 mm in diameter, for patients 05, 08, and 09 in the placebo-treated group. White bars show data from pretreatment OS cycles and black bars show data from post-treatment OS cycles. AMH levels (ng/mL) obtained before and after the Danazol treatment are indicated in the graphs. Total Fol.: Total Follicles; Fol. >12: Follicles larger than 12 mm in diameter; Total oocyt.: Total oocytes; MII oocyt.: MII oocytes; P.: patient.

To test whether Danazol treatment could have effects on long-term fertility, we retrospectively analyzed IVF parameters from other OS cycles that took place at different times (months) before and after the pilot clinical trial. No statistical differences were observed in the AFC after Danazol treatment (Supplementary Fig. S3A). Strikingly, these women (Danazol group) produced oocytes in later OS cycles, after the end of the trial. In the first retrospective cycle, patient 02 had 0.12 ng/mL of AMH and one mature oocyte was collected. For 6 months before the study started (in two OS cycles) the patient did not produce any oocytes. However, patient 02 generated oocytes in OS performed after the trial (months 6 and 7) (Fig. 4A), while her AMH levels were dropping.

Patient 10, whose AMH was close to 0 (<0.01 ng/mL) at the time of recruitment, did not produce oocytes in the 18 months before the study started (two OS cycles), but generated one oocyte 4 months after the end of treatment (Fig. 4B). Patient 12 had higher levels of AMH (2.05 ng/mL) before the clinical trial, yielding mature follicles and MII oocytes (Fig. 4C). At the time of recruitment, her AMH levels were lower (1.2 ng/mL). Interestingly, 3 months after the end of Danazol treatment, this patient had a higher ratio of MII oocytes to mature follicles compared to OS performed before the pilot study (Fig. 4C, D). Indeed, the ratio of MII oocytes to mature follicles obtained after Danazol treatment was higher compared with OS before treatment (Fig. 4D) in the three patients studied.

In the placebo-treated group, the number of oocytes obtained was similar in all OS cycles both before and after the pilot study (Supplementary Fig. S3B–E). The ratio of MII oocytes to mature follicles improved only in one patient (05), while it worsened in two patients (08 and 09) (Fig. 4E).

## Discussion

There is an urgent need in fertility clinics for an efficient strategy to stimulate the ovaries and obtain high-quality oocytes in women with DOR, since patients wish to pass on their own genetic material to their descendants. To this end, we conducted this pilot clinical trial treating women with DOR with Danazol and focusing on fertility parameters and telomere maintenance. It is worth noting that telomerase expression increased cancer incidence in mice at birth,^32^ whereas in adult and old mice, the risk of cancer was similar to controls.^33^

This study shows that telomere maintenance seemed to vary in patients who underwent Danazol treatment. Whether mean TL differences existed in GCs could not be studied as Danazol-treated women had to cancel their oocyte retrieval. TL was similar in the PBMCs (not statistically significant) of the NOR and DOR groups during the visits. However, a higher mean TL in the PBMCs of the Danazol group was observed after 1 month of treatment. Accordingly, also during the first month, the mean percentage of long telomeres was higher, and the percentage of short telomeres was lower. These results were not statistically significant, probably due to the sample size. However, several studies have shown TL increases in PBMCs after Danazol treatment^28,34,35^ possibly due to the ability of steroids to regulate TA^25,26^ and activate telomerase gene expression *in vitro*.^24,27^

After 2 months of treatment, mean TL levels were similar to pretreatment, suggesting that 400 mg per day is not enough to sustain higher mean TL levels for long periods of time, as observed with a dose of 800 mg per day, which sustains higher TL for up to 24 months of treatment and a return to normal TL afterward.^28^ Second, after the first month of Danazol treatment, the number of cells with one TERRA focus decreased and cells with 2 to 4 TERRA foci increased (concurrent with a not-significant lower percentage of short telomeres). Indeed, the presence of short telomeres can induce the generation of TERRAs,^36^ which play critical roles in telomere protection and stability.^9–11^

In fact, TERRAs have been postulated to act as a scaffold to regulate telomerase and other proteins involved in telomere maintenance.^37^ Interestingly, a positive correlation between increased TL—which seems to happen as a result of Danazol treatment^28,34,35^—and TERRA expression has been recently found^38^ and related to embryo quality.^38^

Regarding fertility, our results suggest that Danazol did not benefit IVF outcomes in the first OS performed after the end of Danazol treatment. Actually, it may have had an androgenic effect causing a suppression of the ovary, leading to amenorrhea,^39^ which happened to be temporary, as observed in this study. Interestingly, during the OS cycles performed after the pilot study, we observed that women produced mature oocytes or a higher ratio of mature oocytes with respect to the number of mature follicles, compared to OS performed before the pilot study. It is worth noting that two of the three women treated with Danazol had not produced oocytes for almost 6 months (patient 02) and almost 2 years (patient 10) before the study and showed a very fast decline in AMH levels due to natural aging of the ovary.^1^

The obtention of mature oocytes in OS performed in the months following the end of trial could be due to a direct effect of Danazol on the primordial follicles, which take up to 5 months to mature during folliculogenesis.^40^ Certainly, the long-term effect of Danazol has been previously noted.^28,35,41^

Our main limitation was recruitment, since women undergoing a fertility treatment pursue the shortest time-to-pregnancy possible, especially those with DOR. In addition, the possibility of being randomized for 3 months to a placebo made recruitment even harder, particularly after the pandemic, which had forced patients to delay treatment. Finally, we are aware that the results obtained here may not be representative; thus, more work is needed to understand the possible effects of Danazol on fertility.

## Conclusions

A few months after Danazol treatment, women produced MII oocytes and the ratio of MII oocytes to mature follicles was higher in the three patients treated.

However, more studies with Danazol or other sexual steroids should be conducted to assess their potential effects on fertility.

## Supplementary Material

Supplemental data

Supplemental data

Supplemental data

Supplemental data

## Abbreviations Used

AFC: antral follicle count
AMH: anti-Müllerian Hormone
DAPI: 4′,6-Diamidino-2-phenylindole
DOR: diminished ovarian reserve
EDTA: ethylenediaminetetraacetic acid
EudraCT: European Union Drug Regulating Authorities Clinical Trials Database
FSH: follicle-stimulating hormone
GCs: granulosa cells
GnRH: gonadotropin-releasing hormone
ICSI: intracytoplasmic sperm injection
IVF: *in vitro* fertilization
MII: metaphase II
NOR: normal ovarian reserve
OF: ovarian failure
OS: ovarian stimulation
PBMCs: peripheral blood mononuclear cells
HP-hMG: highly purified human menopausal gonadotropin
HT Q-FISH: high-throughput quantitative fluorescence *in situ* hybridization
RNA FISH: RNA fluorescence *in situ* hybridization
RT: room temperature
SD: standard deviation
TA: telomerase activity
TERRA: telomeric repeat-containing RNA
TL: telomere length
