# Supplementary figures and images for: A Randomized Controlled Intervention Trial with Danazol to Improve Telomeric and Fertility Parameters in Women with Diminished Ovarian Reserve: A Pilot Study
Source: Womens Health Rep (New Rochelle). 2023 Jul 4;4(1):305–18. doi: 10.1089/whr.2023.0013 (PMC10354732; doi:10.1089/whr.2023.0013)

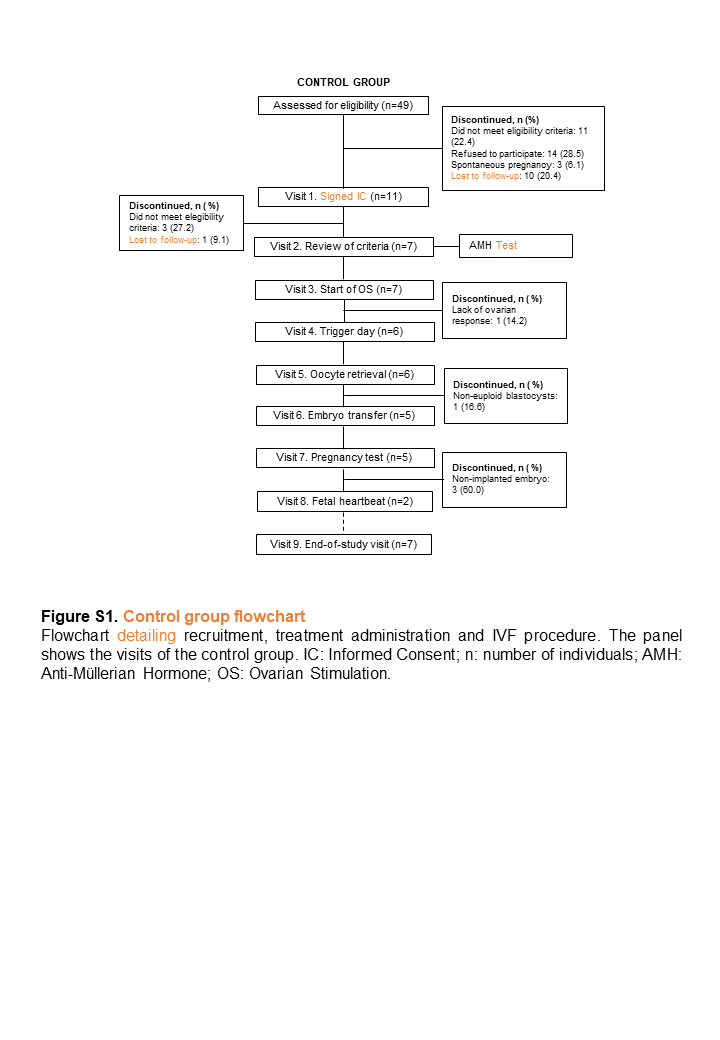

Supplement: Supplemental data [file Suppl_FigureS1.tif]

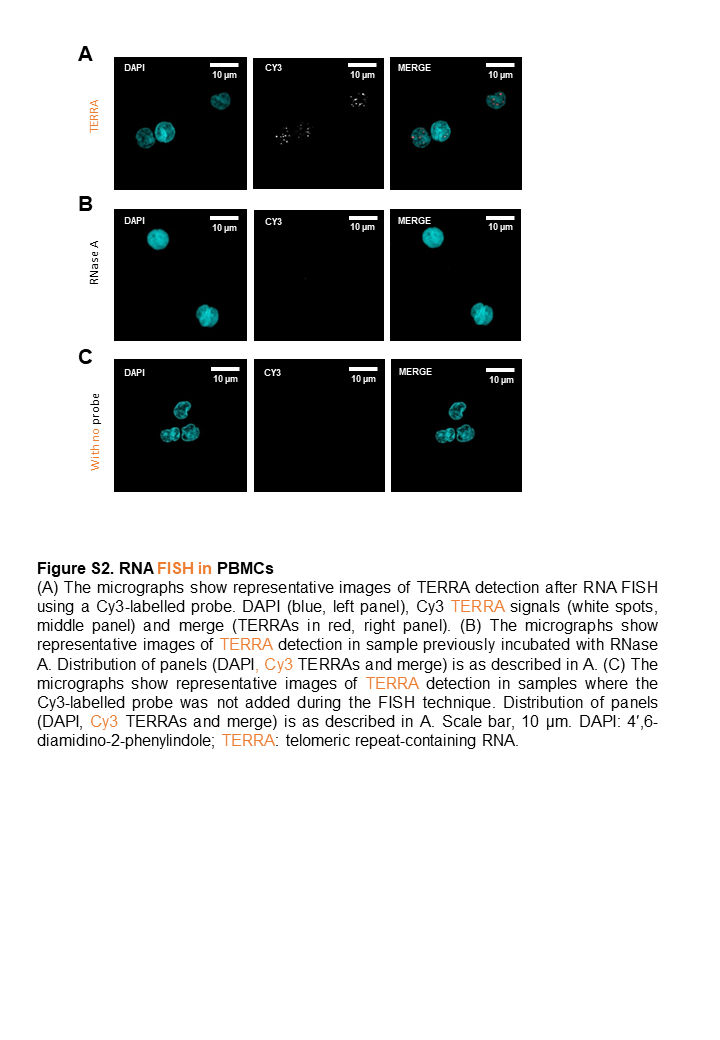

Supplement: Supplemental data [file Suppl_FigureS2.tif]

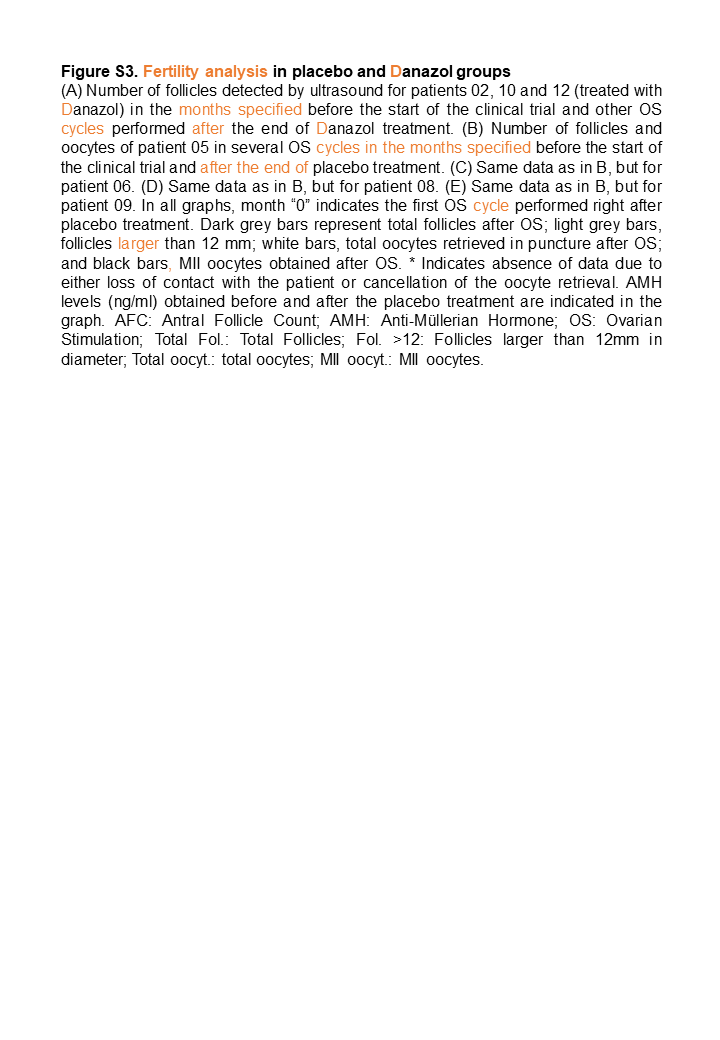

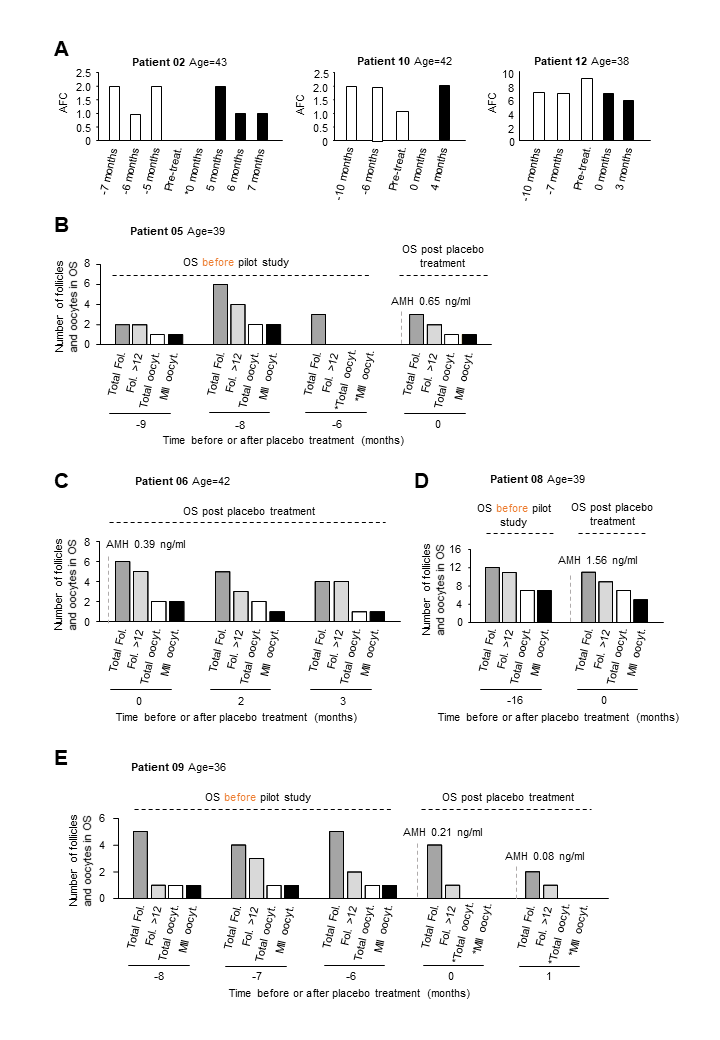

Supplement: Supplemental data [file Suppl_FigureS3.docx]
