# Supplementary material for: A Randomized Controlled Intervention Trial with Danazol to Improve Telomeric and Fertility Parameters in Women with Diminished Ovarian Reserve: A Pilot Study
Source: Womens Health Rep (New Rochelle). 2023 Jul 4;4(1):305–18. doi: 10.1089/whr.2023.0013 (PMC10354732; doi:10.1089/whr.2023.0013)
Supplement: Supplemental data [file Suppl_TableS1.docx]

**Supplementary Table 1. Evaluation of TERRA foci in PBMCs**

|  | **CONTROL**  **GROUP** | **PLACEBO-TREATED GROUP** | **DANAZOL-TREATED**  **GROUP** |
| --- | --- | --- | --- |
| **Pre-treatment, n** | 7 | 4 | 6 |
| Cells with 1 focus (%)  Mean ± SD  Median (Q1-Q3) | 35.5±8.9  36.8 (31.3-40.7) | 35.8±8.5  36.5 (27.2-43.6) | 49.6±18.1  53.5 (31.4-65.6) |
| Cells with 2-4 foci (%)  Mean ± SD  Median (Q1-Q3) | 44.3±3.4  45.3 (42.4-47.4) | 49.0±8.7  49.5 (40.4-57.2) | 39.7±9.1  42.1 (28.9-47.8) |
| Cells with ≥5 foci (%)  Mean ± SD  Median (Q1-Q3) | 20.0±10.9  20.0 (13.1-23.0) | 15.1±4.0  16.3 (10.9-18.0) | 10.8±10.5  7.2 (2.8-20.6) |
| **After one month of treatment, n** | 6* | 4 | 3 |
| Cells with 1 focus (%)  Mean ± SD  Median (Q1-Q3) | 34.8±9.8  34.8 (24.3-45.1) | 41.5±10.1  41.8 (31.6-51.0) | 33.5±4.2  33.3 (29.3-33.8)  ***p* = 0.04 |
| Cells with 2-4 foci (%)  Mean ± SD  Median (Q1-Q3) | 45.4±1.3  45.3 (44.2-46.0) | 46.7±2.2  46.4 (44.8-49.0) | 54.2±3.1  54.8 (50.8-57.1)  ****p* = 0.02 |
| Cells with ≥5 foci (%)  Mean ± SD  Median (Q1-Q3) | 18.2±8.7  18.4 (9.4-25.4) | 11.7±10.7  10.7 (1.8-22.5) | 12.2±3.2  11.2 (9.5-15.8) |
| **After two months of treatment, n** | Not treated | 4 | 3 |
| Cells with 1 focus (%)  Mean ± SD  Median (Q1-Q3) | x | 28.2±8.5  27.1 (20.6-36.8) | 36.6±5.4  38.9 (30.4-40.5)  ****p = 0.01 |
| Cells with 2-4 foci (%)  Mean ± SD  Median (Q1-Q3) | x | 46.7±3.6  47.0 (43.2-49.9) | 50.7±4.8  53.1 (45.1-53.9)  *****p = 0.03 |
| Cells with ≥5 foci (%)  Mean ± SD  Median (Q1-Q3) | x | 25.0±10.0  28.4 (14.4-32.2) | 12.6±5.4  15.6 (6.3-15.9) |
| **After three months of treatment, n** | Not treated | 4 | 5 |
| Cells with 1 focus (%)  Mean ± SD  Median (Q1-Q3) | x | 39.0±13.5  39.0 (25.9-52.1) | 38.5±11.6  31.7 (29.4-51.0) |
| Cells with 2-4 foci (%)  Mean ± SD  Median (Q1-Q3) | x | 44.9±4.2  46.2 (40.6-48.0) | 44.5±4.0  46.2 (40.4-47.7) |
| Cells with ≥5 foci (%)  Mean ± SD  Median (Q1-Q3) | x | 16.0±9.8  14.7 (7.1-26.0) | 16.9±8.3  21.2 (8.6-23.1) |

Evaluation of the percentages of TERRA foci in PBMCs of the control, placebo-treated and danazol-treated groups. n indicates the number of individuals. Normality was calculated using the Shapiro-Wilk test. *Patients in the control group had no treatment; these values were measured on their 5^th^ visit. **p-value between the mean percentage of 1 focus pre-treatment and after one month of treatment in the danazol-treated group. ***p-value between the mean percentage of 2-4 foci pre-treatment and after one month of treatment in the danazol-treated group. ****p-value between the mean percentage of 1 focus pre-treatment and after two months of treatment in the danazol-treated group. *****p-value between the mean percentage of 2-4 foci pre-treatment and after two months of treatment in the danazol-treated group.
